# Neural correlates of top‐down modulation of haptic shape versus roughness perception

**DOI:** 10.1002/hbm.24764

**Published:** 2019-08-20

**Authors:** Stefanie Mueller, Benjamin de Haas, Anna Metzger, Knut Drewing, Katja Fiehler

**Affiliations:** ^1^ Department of Experimental Psychology Justus Liebig University Giessen Germany; ^2^ Leibniz Institute of Psychology Information (ZPID) Trier Germany; ^3^ Center for Mind, Brain, and Behavior (CMBB) Marburg University and Justus Liebig University Giessen Germany

**Keywords:** fMRI, haptic perception, roughness, shape, touch

## Abstract

Exploring an object's shape by touch also renders information about its surface roughness. It has been suggested that shape and roughness are processed distinctly in the brain, a result based on comparing brain activation when exploring objects that differed in one of these features. To investigate the neural mechanisms of top‐down control on haptic perception of shape and roughness, we presented the same multidimensional objects but varied the relevance of each feature. Specifically, participants explored two objects that varied in shape (oblongness of cuboids) and surface roughness. They either had to compare the shape or the roughness in an alternative‐forced‐choice‐task. Moreover, we examined whether the activation strength of the identified brain regions as measured by functional magnetic resonance imaging (fMRI) can predict the behavioral performance in the haptic discrimination task. We observed a widespread network of activation for shape and roughness perception comprising bilateral precentral and postcentral gyrus, cerebellum, and insula. Task‐relevance of the object's shape increased activation in the right supramarginal gyrus (SMG/BA 40) and the right precentral gyrus (PreCG/BA 44) suggesting that activation in these areas does not merely reflect stimulus‐driven processes, such as exploring shape, but also entails top‐down controlled processes driven by task‐relevance. Moreover, the strength of the SMG/PreCG activation predicted individual performance in the shape but not in the roughness discrimination task. No activation was found for the reversed contrast (roughness > shape). We conclude that macrogeometric properties, such as shape, can be modulated by top‐down mechanisms whereas roughness, a microgeometric feature, seems to be processed automatically.

## 1 INTRODUCTION

When we explore the shape of an object with our hand, we inevitably gather information about the surface of this object, for example, the roughness, as well. However, for the task, at hand roughness is irrelevant. Here we propose that shape and roughness are processed differently depending on their momentary task‐relevance.

Using behavioral psychophysics, it has been demonstrated that top‐down mechanisms such as feature‐ or location‐based attention can alter performance in haptic perception tasks. Psychophysical experiments utilizing cueing paradigms demonstrated improved performance when attention was preallocated to the body location at which the target appeared or to the feature that had to be judged (Gomez‐Ramirez, Hysaj, & Niebur, 2016). Conversely, invalid feature and location cues have been shown to decrease performance in haptic perception tasks (Forster & Eimer, 2005; Sinclair, 2000). A recent study by our group (Metzger, Mueller, Fiehler, & Drewing, 2019) employed a task in which participants explored two successive stimuli that both had a defined shape and roughness. After exploration, either the shape or roughness of the two stimuli had to be compared. Importantly, the occurrence of either task was varied such that participants expected to judge one feature more frequently than the other. If the task was unexpected, that is, the roughness had to be judged when participants expected the shape task or vice versa, performance deteriorated.

Haptic features are commonly subdivided into macrogeometric and microgeometric features. A macrogeometric feature refers to global properties of the object such as shape or size, whereas microgeometric properties refer to small, local surface deviations (Roland & Mortensen, 1987). Neuroimaging research suggests that the haptic processing of macrogeometric and microgeometric features is (at least partly) associated with different brain areas. For example, the postcentral sulcus and the postcentral gyrus (PoCS: Peltier et al., 2007; Stilla & Sathian, 2008; PoCG: O'Sullivan et al., 1994), the intraparietal sulcus (IPS; Peltier et al., 2007; Podrebarac, Goodale, & Snow, 2014; Stilla & Sathian, 2008; Roland, O'Sullivan, & Kawashima, 1998), and the lateral occipital cortex (LOC; Peltier et al., 2007; Stilla & Sathian, 2008; Amedi, Malach, Hendler, Peled, & Zohary, 2001) showed stronger activation for the processing of shape than of texture. The processing of texture as compared to shape revealed more mixed results. However, the insula and the adjacent parietal operculum (PO) have been reported across multiple studies (Ledberg, O'Sullivan, Kinomura, & Roland, 1995; Stilla & Sathian, 2008; Roland et al., 1998; posterior collateral sulcus: Podrebarac et al., 2014).

The functional role of these brain areas has been further specified by studies focusing on the processing of either the macrogeometry or the microgeometry of an object, rather than directly comparing the two. For example, LOC has been identified to contribute to haptic object recognition when the palpation of everyday objects was contrasted against that of (complex) nonsense objects (Reed, Shoham, & Halgren, 2004) and the IPS was more active when manually discriminating complex object shapes than low‐level tactile properties such as the temperature of two spheres (Rojas‐Hortelano, Concha, & de Lafuente, 2014). Thus, LOC and IPS seem to be specifically involved in higher order processing of haptic shape. Regarding the microgeometric domain, participants were asked to either judge the perceived spatial density or the perceived roughness of the same set of dot patterns (Eck et al., 2016). The perception of the two features relates differently to the physical property of the interdot spacing. While the perceived spatial density decreases linearly when the spaces between dots are increased, roughness perception follows a more complex function. The difference in complexity could be decoded on the neuronal level. Early somatosensory cortices were recruited in the processing of both features while only roughness information could be decoded in higher‐order cortices such as the insula, the PO, and the ventral temporal cortex.

Previous studies mainly examined the neural correlates of haptic perception of shape and roughness by separately varying the macro‐ and microgeometric features on different objects (e.g., Bodegård et al., 2001). This approach is useful to identify segregated processing related to different sensory inputs, especially on lower levels of the sensory hierarchy. However, it does not allow dissociating between sensory‐driven, bottom‐up and top‐down modulated processes. Moreover, it is potentially confounded by differences in the exploration movements and/or the types of stimuli used. The present study aimed to investigate how top‐down control, varied by task‐relevance, changes the cortical processing of two complex somatosensory features, shape, and roughness, and how this relates to the individual behavioral performance. We controlled for stimulus‐driven activations by keeping the exploratory procedure and stimulus type constant across conditions. To identify top‐down modulated versus stimulus‐driven activations, we created multidimensional objects varying in both shape and roughness. Participants were trained to haptically explore these objects by the same lateral contour‐following movement, employing the exploratory procedure that is optimal to estimate the elongation of an object (contour following) and to estimate the roughness of its surface (lateral motion, Lederman & Klatzky, 1987). They explored the same objects under two task instructions: Either to compare the shape of two successive objects (Which object is longer?) or their roughness (Which object is rougher?). Thus, we manipulated the task‐relevance of each feature. The object features were parametrically varied which allowed us to determine the individual discrimination thresholds which were then related to the brain activation.

## 2 METHODS

### 2.1 Participants

A total number of 25 right‐handed individuals took part in this study. In the course of the analyses, the datasets of four participants were removed: One because of movement artifacts, three due to behavioral performance criteria (see section 2.6). The remaining sample of 21 participants (11 females) was aged between 21 and 34 years (mean [M] = 25.33 years; standard deviation [*SD*] = 3.44 years). All participants were healthy and reported no history of neurological or psychiatric disorders. Written informed consent was collected before participation in accordance with the guidelines approved by the local ethics committee of the Department of Psychology and Sports Sciences of the Justus‐Liebig University Giessen (Lokale Ethik‐Kommission des Fachbereichs 06, LEK‐FB06) and in line with the Declaration of Helsinki (World Medical Association, 2013). Participants received either monetary compensation (8€/hr for the behavioral training, 10€/hr for the fMRI scanning) or course credit.

### 2.2 Stimuli

**Figure 1:**
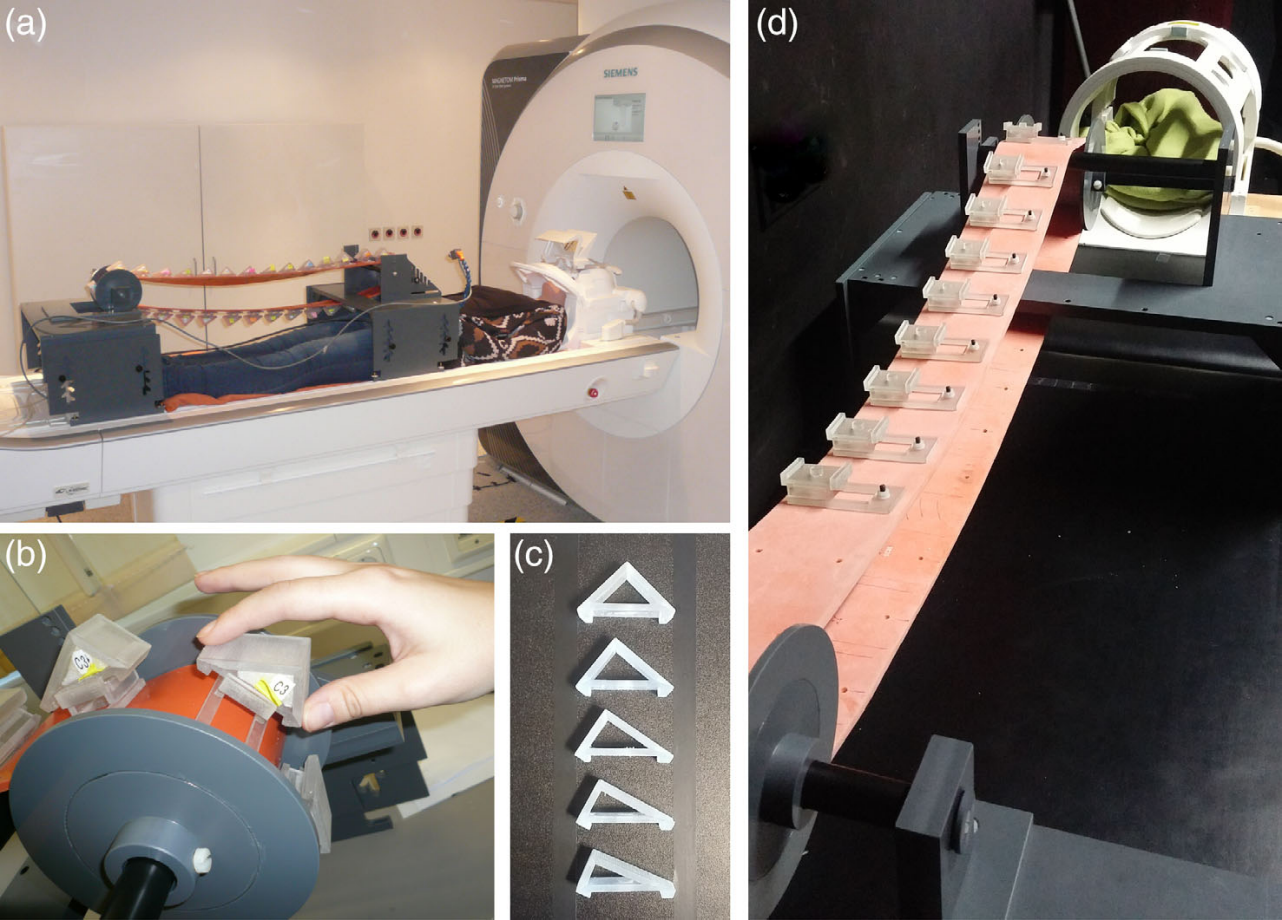
Apparatus and objects. (a) The apparatus was placed over the participant in the scanner so that the object presented in a given trial could be comfortably reached with the right hand. Participants viewed the visually presented task instruction through a mirror mounted on the head coil. (b) Participants explored the (pseudo‐)cuboids by grasping the outer edges of the cuboid with thumb and index and then simultaneously moving the fingers until they met on the top edge. (c) Shape and roughness of the objects were varied on five levels. Here, the five levels of oblongness are shown. (d) Objects were mounted on a conveyer belt which was manually operated by the experimenter during scanning [Color figure can be viewed at http://wileyonlinelibrary.com]

The stimuli were cuboids that differed in their shape (oblongness) and roughness. They were produced by 3D printing (Object30Pro, Stratasys, material VeroClear, nominal resolution 600–1,600 dpi). With regard to shape, the cuboids had the same diagonal length (*d* = 56.57 mm) and width (*w* = 30 mm) but differed in the relative length of their sides such that five different levels of oblongness were created (lengths of the shorter/longer side 40.0/40.0, 35.6/44.0, 31.2/47.2, 26.8/49.8, 22.4/51.9 mm; see Figure 1). Stimuli were always presented in the same orientation, that is, with the shorter side facing towards the participant (Figure 1b). The surface of each cuboid possessed a certain roughness. Roughness was defined as one‐dimensional (1D) square‐wave function with an amplitude of 0.3 mm and was varied on five levels by groove widths of 0.25, 0.34, 0.42, 0.51, or 0.59 mm, respectively. Ridge width always equaled groove width. In every trial, a standard (31.2 mm length for shape, 0.42 mm groove width for roughness) had to be judged against a comparison stimulus (1 of the 5 levels of roughness/shape).

Since participants only explored half of the object (one short and one long side), the objects used in the experiment were printed as pseudo‐cuboids, that is, cuboids that were “cut” 2 mm below their diagonal. This rendered half of a cuboid plus prolongations of a height of 2 mm at the edges (Figure 1C). The prolongations ensured that participants still had the impression of grasping a full cuboid. For more information on how the stimuli were created and how the different levels of the stimuli were determined, see Metzger et al. (2019), the behavioral predecessor of the present study.

The exploration movement was demonstrated by the experimenter before the start of the experiment using complete cuboids. After this demonstration, participants always performed the exploration task without vision of the objects and objects were covered when in view of the participant. In particular, participants were instructed to either judge the shape or the roughness of two successively presented objects. In the shape task, participants were asked to indicate which object felt longer. In the roughness task, participants indicated which of the two objects felt rougher.

In a fully randomized design, 25 unique objects are possible by the combination of the five roughness and the five shape levels and multiple exemplars (ideally all 25 for every trial in a block and in a session = 25 × 5 × 3 = 375 objects) would be needed to ensure a reasonably rapid presentation of trials. Since printing this saturated set of objects would have been a long and costly process, we opted for a more economical solution and limited the number of objects by producing six sets of objects that were predefined as follows. Each set consisted of 10 objects corresponding to five trials (one trial = two objects). Each trial contained both the roughness and the shape standard (not necessarily on the same object) and each set contained all five possible comparisons of the standard against the five comparison levels for both features/dimensions. Adhering to these specifications, the objects of each set were pseudorandomly determined and then printed. Table 1 lists the parameters of one set of objects as an example.

**Table 1:** Set 1 (out of six sets) of objects presented in one exploration block

Object		Object	
Shape (longer side)	Roughness	Shape (longer side)	Roughness
22.40 (51.95)	0.59	31.20 (47.19)	0.42
26.80 (49.82)	0.42	31.20 (47.19)	0.42
31.20 (47.19)	0.34	40.00 (40.00)	0.42
31.20 (47.19)	0.42	31.20 (47.19)	0.51
35.60 (43.96)	0.42	31.20 (47.19)	0.25

*Note*. The two objects in every row were presented in one trial. The order of the two objects in one trial was randomized as was the order of trials across one set/block. Each trial/row contains the shape standard (31.20) and the roughness standard (0.42). Each set contained all five comparisons in the shape (22.40, 26.80, 31.20, 35.60, and 40.00) and the roughness dimension (0.25, 0.34, 0.42, 0.51, and 0.59).

### 2.3 Apparatus

In order to allow for quick successive object presentations, all the stimuli presented in one session were mounted on a conveyer belt of rubber (length = 208 cm) running between two cylinders. The cylinders were mounted on separate tables which could be fixated on the scanner bed (see Figure 1a). One cylinder was at waist level of the participant, who lay under the apparatus. When a stimulus was over that cylinder, facing the participants, they could comfortably grasp this stimulus with the right hand (see Figure 1b). The other cylinder was at foot‐level and connected with a crank handle by which the experimenter manually turned the conveyor belt. The conveyor belt carried 30 mounts (a total of three sets of objects) on which the objects were attached (see Figure 1d). The apparatus was partly moved inside the scanner bore during scanning.

### 2.4 Procedure

Participants successively explored two objects with their right hand and then reported which of the two objects felt longer (shape task) or rougher (roughness task).

The experiment comprised a behavioral training and an MRI session. The behavioral training was conducted outside of the scanner and served to familiarize the participants with the task and the timing of the experiment. The experimenter explained the task and showed how to perform the exploration movement on two (complete) cuboids. Specifically, participants were instructed to grasp the two edges of the diagonal of the cuboid with their thumb and index of the right hand and to move them in one stroking movement across the surfaces until the thumb and index finger met on the upper edge (see Figure 1b). It was emphasized (and later ensured during the practice trials) to always perform the same exploration movement irrespective of the task. Once the task and the exploration movement were understood, the participant lay down on a stretcher and the apparatus was positioned above them. The participant completed some practice trials until the task could be performed smoothly in the required time. The behavioral training took about half an hour.

The MRI session was conducted up to 2 days after the training and took about 1.5 hr in total. Before the image acquisition started, participants completed a few more practice trials to remind them of the procedure. The presentation of visual and auditory stimuli was controlled by Presentation® software (Version 17.2, http://www.neurobs.com).

The MRI session was conducted in two runs with a break in between. Each run took approximately 4 min. During the break, structural images and a second field map were acquired. The experimenter changed the objects on the conveyor belt during anatomical scanning.

**Figure 2:**
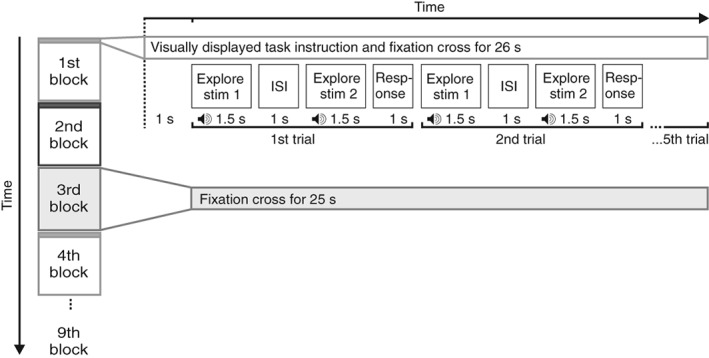
Schematic time course of one experimental run. On the left, the sequence of blocks is shown. Unfilled boxes denote exploration blocks in which participants explored the objects with their right hand; the filled box denotes a baseline block. The task of the first block (shape/roughness) was counterbalanced across participants and determined the subsequent order of tasks. The third, sixth, and ninth block were always baseline blocks. The right section provides details about the time courses within block types. The exploration blocks started with the visual presentation of the task instruction (judge shape/roughness). After 1 s, a tone signaled the onset and duration of the exploration phase (1.5 s) for the first object. After a short interstimulus interval (1 s ISI), a tone signaled the exploration phase of the second object. The response had to be given with the left hand within 1 s after the second exploration phase ended. Then, the next trial started. In the baseline blocks, a fixation cross was presented on a screen behind the scanner bore for 25 s. Participants were instructed to fixate the cross and to rest their hands

Each run consisted of nine blocks (three each for shape, roughness, and baseline) in a fixed order of two exploration blocks (see Figure 2, unfilled boxes in the left column) followed by a baseline block (see Figure 2, filled box in the left column) which was repeated three times, that is, task 1, task 2, baseline, task 1, task 2, baseline, task 1, task 2, baseline. The order of the exploration blocks (shape/roughness or roughness/shape) was counterbalanced across participants. One exploration block corresponded to one set of objects and consisted of five trials (two objects per trial). The order of sets, the order of trials within each set and the order of standard/comparison stimulus within each trial was randomized for every participant. Within each session, a given set was presented twice: Once under the instruction to judge the shape, and once under the instruction to judge the roughness of the (pseudo‐) cuboids.

The shape and the roughness blocks started with the presentation of the respective instruction and then five trials of the instructed task were performed. The instruction, together with a cross to fixate, was displayed continuously until the fifth trial ended. Then, the instruction of the other task was presented and five trials of this task were performed.

Each trial of the exploration blocks started with the presentation of a tone for 1.5 s. The tone marked the timeframe for the exploration of the first object. Then, an interstimulus interval of 1 s followed in which the experimenter operated the crank handle presenting the second object. Again, a tone of 1.5 s was presented during which the second object was explored. Afterward, the participant was given 1 s to respond by pressing a button with their left hand. If the first object felt longer/rougher, participants were instructed to press the left button otherwise they pressed the right button on the response pad. During this time, the experimenter turned the handle to present the first object of the next trial.

The haptic exploration time was set to 1.5 s based on the results of a behavioral study using the same stimuli in a similar task (Metzger et al., 2019). In this study, participants were instructed to manually explore each object once without time restriction. We analyzed movement parameters and revealed a mean exploration time of 1.318 s per object for the expected shape task and 1.298 s for the expected roughness task. Thus, an exploration time of 1.5 s provided participants enough time to explore the objects and make reliable judgments about their shape and roughness. During the training session and practice trials, we further ensured that participants were able to keep up with the pace of the stimulus presentation.

The baseline blocks consisted of the presentation of the fixation cross for 25 s during which participants rested their hands. During the baseline and the exploration blocks, participants were instructed to remain gaze at the fixation cross.

An MRI‐compatible camera (12m‐i, MRC Systems GmbH, controlled via ViewPoint Software, Arrington Research) directed at the participant's right hand and the current object was used for online control of the participants’ compliance with instructions, that is, use of trained exploration movement in exploration blocks, no touching of objects during baseline blocks.

### 2.5 Data acquisition

The MRI session was carried out on a 3‐Tesla imaging system (Siemens Prisma) at the Bender Institute of Neuroimaging (BION) at Giessen University. Participants’ heads were placed in a 64‐channel head coil and stabilized by foam padding. A mirror mounted on the head coil allowed viewing of an MRI‐compatible monitor (BOLDscreen 32, Cambridge Research Systems Ltd., 31.55″ diagonal, 1,920 × 1,080 pixel) that was located behind the scanner. During functional scans, the respiration and heart rate were measured by a respiration belt and a finger clip attached to the left ring finger.

Functional images covered the whole brain and were obtained by using an echo‐planar imaging sequence (EPI) with an echo time (TE) of 30 ms and a repetition time (TR) of 2,200 ms. Further parameters for obtaining functional data were set as follows: Field of view (FoV) = 214 × 214 mm, in‐plane resolution = 2.97 mm × 2.97 mm, 40 sagittal slices (descending) with a thickness of 3 mm (slice spacing = 3.75 mm) and a distance factor of 25%, flip angle (FA) = 79°, acceleration factor = 2.

High‐resolution anatomical images were obtained using a T1‐weighted magnetization‐prepared rapid acquisition gradient‐echo (MPRAGE) sequence with the following scan parameters: FoV = 240 × 240 mm, TE = 3.53 ms, TR = 1880 ms, inversion time = 949 ms, in‐plane resolution = 0.94 mm × 0.94 mm, number of slices = 176, slice thickness = 0.94 mm (slice spacing = 0.94 mm), flip angle (FA) = 8°.

Perturbations of the magnetic field were accounted for by measuring a field map for each of the two sessions with the following scan parameters: FoV = 220 × 220 mm, TE (1) = 10 ms, TE (2) = 12.46 ms, TR = 1,000 ms, in‐plane resolution = 2.0 × 2.0 mm, slice thickness = 3.0 mm (slice spacing = 3.75 mm), number of slices = 40 (transversal), FA = 90°. Per participant, 230 volumes of functional data were acquired (115 per run).

### 2.6 Data analysis

#### 2.6.1 Behavioral data analysis

We determined the percentage of correct responses (excluding trials in which the shape/roughness of the standard and the comparison were equal) for each participant in both tasks averaged across sessions. If the performance of a participant was below 60% in both tasks or was below or equal to 50% in one task, the participant was excluded, which was the case for three out of 25 participants. Another participant was excluded due to movement artifacts (see section 2.6.2). On average, the final sample of 21 participants responded correctly in 81.94% (standard error of the mean [*SEM*] = 2.64%) of the trials in the roughness task and in 72.22% (*SEM* = 2.23%) of the trials in the shape task. Participants performed better when comparing the roughness of two objects than when comparing their shape, as determined by a paired *t*‐test (*t*_20_ = −3.45, *p* = .003).

We further fitted psychometric functions (cumulative Gaussian functions) to the responses of each participant in each task using psignifit 3.0 (Schütt et al., 2016). Since each fit was based on very few observations (six repetitions for each of the five stimulus levels), the lapse and guess parameter were fixed to zero. The 50% threshold (mean across participants in the shape and roughness task: 30.27 and 0.44 mm) and the 84% difference threshold (*SD*; mean across participants in the shape and roughness task: 8.42 mm and 0.12 mm) were determined by fitting a sigmoid function to the data. The 50% threshold indicates the length/roughness at which the two stimuli cannot be discriminated by the participant (50% chance of saying first or second stimulus was rougher/longer). The 84% difference threshold corresponds to the difference between the mean ± 1 *SD* in the standardized normal distribution and is a standard parameter used to evaluate the discrimination performance in psychometric fitting (Helbig & Ernst, 2007). Thresholds for individual participants and exemplary psychometric functions are provided in the Supporting Information (see Figure S1).

#### 2.6.2 FMRI data analysis

Data were preprocessed and analyzed with SPM12 (Statistical Parametric Mapping, Wellcome Department of Imaging Neuroscience, University College London, UK) running in MATLAB (R2016a) and selected tools from FSL (FMRIB's Software Library, Oxford, UK; http://www.fmrib.ox.ac.uk/fsl). The raw data and the output of each of the different preprocessing steps were visually checked for artifacts. As mentioned above, one participant was excluded due to severe movement artifacts during functional and structural scanning. Significant activations were anatomically labeled using the Harvard Oxford Sub/Cortical (Bakker, Tiesinga, & Kötter, 2015; Desikan et al., 2006; Frazier et al., 2005; Goldstein et al., 2007; Makris et al., 2006) and the Juelich Histological Atlas (Eickhoff et al., 2005, 2007; Eickhoff, Heim, Zilles, & Amunts, 2006) as implemented in FSLview.

##### Preprocessing

Functional data were realigned and unwrapped using the voxel displacement maps generated from the field maps. Then, the functional images were coregistered with the high‐resolution structural image of the respective participant. Slice time correction was performed before normalization to the MNI 152 template. The normalized data were spatially smoothed using a 5 mm Gaussian kernel. The fMRI time series of every participant were further screened with FACT (fMRI Artifact Correction Tool), an FSL tool comparing consecutive volumes, thereby identifying possible artifacts (e.g., caused by motion) which were entered as covariates of no interest in the first‐level analysis of the respective participant. Physiological data (heart rate, respiration) were extracted by a custom‐written tool (BION_physex, written by Bertram Walter), then entered into the TAPAS PhysIO Toolbox (running in MATLAB) which created 20 regressors for physiological noise correction based on a variety of noise models (Kasper et al., 2017 for detailed information) by application of the default settings of the toolbox. Particularly, out of these 20 regressors, six relate to the phases of pulse modeled by sinus and cosinus functions plus two harmonic functions respectively, eight describe the phases of respiration modeled by sinus and cosinus functions plus three harmonic functions, respectively, four describe the interaction of pulse and respiration (sinus and cosinus functions of pulse multiplied with sinus and cosinus functions of respiration, read more: Glover, Li, & Ress, 2000), one describes the respiratory volume per time (Birn, Smith, Jones, & Bandettini, 2008), and one describes the heart rate variability (Chang, Cunningham, & Glover, 2009). The 20 nuisance regressors were included in the first‐level analysis of the respective participant. For 6 out of 21 participants, the heart rate data was not recorded due to human error, resulting in nine nuisance regressors for these participants.

##### General linear models

Boxcar regressors were specified for the blocks of the shape and roughness condition, for the baseline condition, and for the button press of the response. The boxcar functions of the shape and roughness condition comprised the 25 s from the beginning of the first trial of a block until the end of the last (fifth) trial of that block. The boxcar function for the response was defined with a fixed duration of 0.1 s at the time the button press was registered. Each of the four regressors was then convolved with the canonical hemodynamic response function as implemented in SPM. Additionally, the six realignment parameters created by the rigid‐body transformation of SPM's motion‐correction procedure, the artifact regressors created by FACT, and the regressors for the correction of physiological noise (where available) entered the GLM analysis. The two runs of data collection were implemented as separate runs in a single GLM per participant. The time series for each voxel was high‐pass filtered with a cut‐off of 1/128 Hz, that is, a discrete cosine basis set was included in the first‐level design matrices to model low‐level signal drifts. The two task conditions were each contrasted against the baseline condition (shape > baseline, roughness > baseline) and against each other (shape > roughness, roughness > shape) resulting in four contrast images per participant.

As a solution to the problem of multiple testing, we opted for the nonparametric approach of permutation tests to identify clusters of supra‐threshold voxels as implemented by the SnPM13 toolbox (http://warwick.ac.uk/tenichols/snpm; Nichols & Holmes, 2002) for SPM. This approach uses the General Linear Model to construct pseudo‐*t*‐statistic images, which are then assessed for significance by comparison to an empirically estimated null distribution derived from the data. Given the null hypothesis, the condition labels are interchangeable, that is, contrast images between conditions would be centered on zero. Thus, the null distribution of any statistic can be empirically derived by permuting the labels of two conditions (when comparing two groups) or by permuting, that is, flipping, the sign of contrast images (in case of one contrast image per subject) many times. Comparing the observed data against this empirical null distribution provides a probability estimate of the observed parameter value under the null hypothesis. Or more precisely: This probability estimate (i.e., the *p*‐value) refers to the proportion of values drawn with permuted labels/flipped signs that are greater or equal to the observed parameter. The parameter value can refer to the contrast statistic of a single voxel or the extent of a cluster of contiguous voxels crossing a threshold (defined a priori). For each permutation, the *maximum* value of the corresponding parameter is determined across the whole image and registered for the null distribution. This approach thus allows family‐wise error corrected statistical inference (Poldrack, Mumford, & Nichols, 2011). Detailed information describing permutation testing is provided by Nichols and Holmes (2002).

Using the SnPM toolbox, we performed one nonparametric test for each of the four contrasts (shape > baseline, roughness > baseline, shape > roughness, roughness > shape) by setting the parameters as follows: The design was specified for multiple subjects and one‐sample *t*‐tests on contrast images testing whether the activation differences reflected in the first‐level contrasts significantly exceeded zero across subjects. For each contrast, the signs of the respective first‐level contrast images were permutated 5,000 times (which is the default setting).

When comparing the two exploration conditions against each other (shape > roughness, roughness > shape) we used cluster‐wise inference to correct for multiple comparisons, with an alpha‐level of *p* < .05 (FWE) and a cluster‐forming threshold of *p* < .0001, resulting in a critical suprathreshold cluster size (STCS) of >14 voxels. When examining the exploration conditions against the baseline we opted for a conservative threshold of *p* < .05 FWE on the voxel‐level, with an additional cluster‐size requirement of >14 voxels.

#### 2.6.3 Correlations between brain activation and behavioral data

We correlated behavioral performance with brain activation across participants. Behavioral performance was defined as the 84% difference threshold in the shape/roughness task as estimated by the psychometric fits. As a measure of brain activation, we first defined regions of interest (ROI) based on the contrast between the shape and the roughness task. Within each of these ROIs, we extracted the median *t*‐values from the contrast images shape/roughness > baseline for every participant. The median *t*‐values were used as proxy for brain activation. Pearson correlation coefficients are reported. The correlation coefficients between the shape and the roughness task were compared using the Pearson–Filon statistic for correlated but nonoverlapping correlations (Raghunathan, Rosenthal, & Rubin, 1996).

## 3 RESULTS

### 3.1 Baseline contrasts of shape and roughness

**Figure 3:**
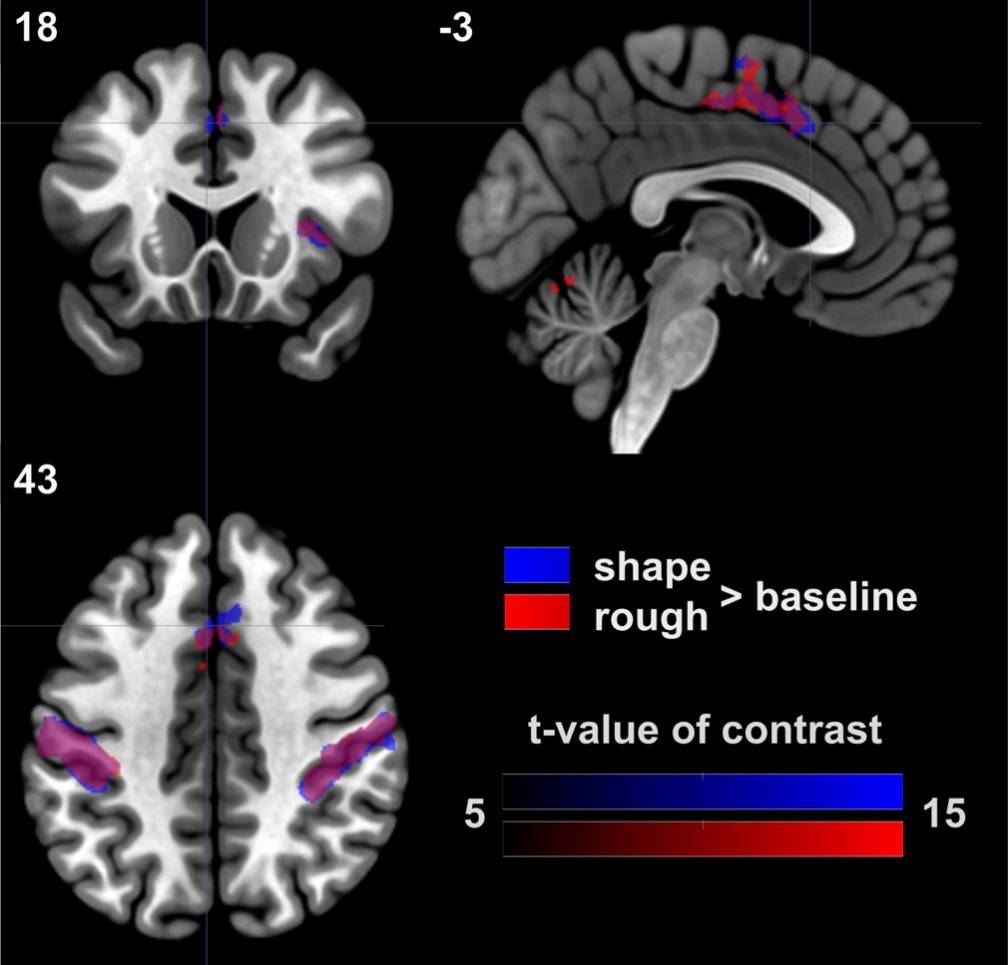
Contrasts against baseline. The contrasts of shape > baseline and roughness > baseline yielded common activations reflecting a widespread network of haptic shape and roughness perception. Large, bilateral activations were found in postcentral gyrus, precentral gyrus, cerebellum, and insula. Left‐hemispheric activations were found in the lateral occipital cortex and the thalamus. An activation in the right inferior temporal gyrus and a cluster stretching from the right paracingulate gyrus to the left supplementary motor cortex were identified. The contrast of shape > baseline further yielded activations in the right superior parietal lobule and in two clusters which overlapped with the left planum temporale and spread to the postcentral gyrus, the Heschl's gyrus, and the parietal operculum [Color figure can be viewed at http://wileyonlinelibrary.com]

Contrasting each of the exploration conditions against the baseline condition revealed a widespread network involved in haptic exploration and processing as shown in Figure 3 (also see Table 2). Strikingly, significant areas were very similar between the shape and the roughness condition, comprising PoCG, precentral gyrus (PreCG), insular cortex and cerebellum bilaterally, the LOC and thalamus in the left hemisphere, the inferior temporal gyrus in the right hemisphere as well as a cluster stretching from the right paracingulate gyrus to the left supplementary motor cortex (SMA). Activations for the shape task were found in the right superior parietal lobule (SPL) and in two clusters overlapping in the left planum temporale, one extending into the inferior PoCG and Heschl's gyrus and the other one extending into the PO.

**Table 2:** Baseline contrasts

	Shape > baseline				Roughness > baseline			
	MNI*x y z*	*k*	*T*	*p*	MNI	*k*	*t*	*p*
*Significant left‐hemispheric voxel activations for baseline contrasts*								
Precentral and postcentral gyrus	−32 −24 50	2027	13.76	<.001	−36 −38 64	2,357	15.24	<.001
	−54 −20 50		13.01	<.001	−44 −14 56		13.52	<.001
	−44 −14 56		12.74	<.001	−52 −20 50		13.23	<.001
Postcentral gyrus, planum temporale, and Heschl's gyrus	−62 −18 16	57	9.11	.001				
	−60 −16 8		7.76	.017				
	−50 −18 10		7.50	.029				
Lateral occipital cortex	−50 −64 −6	54	10.58	<.001	−52 −68 −8	25	8.94	.002
					−48 −62 −4		7.45	.032
Precentral gyrus	−58 0 34	147	10.56	<.001	−58 0 34	176	11.05	<.001
	−60 4 22		8.16	0.008	−60 4 22		9.62	.001
	−58 10 30		7.87	0.014	−58 10 30		8.40	.005
Cerebellum	−24 −52 −24	211	10.39	<.001	−26 −54 −24	257	10.69	<.001
	−26 −64 −24		9.92	<.001	−26 −64 −24		9.86	<.001
	−18 −72 −22		9.11	.001	−32 −70 −22		9.20	.001
Cerebellum	−14 −70 −46	36	7.97	.011	−20 −64 −50	51	7.33	.040
	−24 −72 −52		7.90	.013	−14 −70 −46		7.22	.051
					−20 −72 −52		6.97	.085
Thalamus	−14 −20 8	49	9.71	<.001	−10 −18 6	60	9.56	.001
Right paracingulate gyrus and left	4 14 46	292	9.52	.001	−4 −8 48	342	8.72	.003
Supplementary motor cortex	−6 −10 50		8.50	.004	−4 −16 50		8.68	.003
	8 24 42		8.21	.007	4 14 46		8.65	.003
Insular cortex	−38 −4 12	17	8.30	.006	−36 −4 12	31	9.19	.001
Parietal operculum and planum	−44 −28 20	49	8.68	.003				
Temporale	−50 −26 14		7.56	.026				
	−48 −36 14		7.37	.037				
*Significant right‐hemispheric voxel activations for baseline contrasts*								
Postcentral gyrus and supramarginal gyrus	48 –26 46	989	13.67	<.001	48 –26 48	1,011	14.41	<.001
	58 –18 38		13.62	<.001	56 –18 38		12.28	<.001
	46 –34 52		13.00	<.001	64 –14 22		11.37	<.001
Cerebellum	22 –48 −24	670	12.40	<.001	22 –50 −24	800	13.75	<.001
	8 –64 −14		11.91	<.001	16 –54 –16		12.95	<.001
	16 –54 −22		11.81	<.001	6 –64 −14		10.72	<.001
Cerebellum	20 –60 −50	364	10.90	<.001	6 –68 −38	430	11.81	<.001
	6 –68 −36		9.84	<.001	20 –60 −50		10.56	<.001
	10 –74 −48		9.57	.001	14 –68 −46		10.28	<.001
Precentral gyrus and inferior frontal gyrus, pars opercularis	56 10 28	184	10.44	<.001	56 10 26	176	9.53	.001
	60 12 14		8.29	.006	62 12 20		9.23	.001
	62 12 22		8.10	.009	48 8 28		6.58	.192
Precentral gyrus and superior frontal gyrus	28 –6 50	125	10.18	<.001	28 –6 50	105	10.59	<.001
	22 –10 58		8.09	.009	22 –10 58		8.47	.004
	22 4 52		7.18	.054				
Precentral gyrus	40 –10 58	26	10.17	<.001	40 –10 58	36	10.76	<.001
Inferior temporal gyrus	50 –58 −8	30	9.67	.001	50 –58 −8	15	8.62	.003
Insular cortex	40 –2 10	24	9.02	.002	38 –2 12	30	9.10	.001
Anterior insular cortex	34 20 6	45	8.07	.009	34 20 6	36	7.90	<.001
Superior parietal lobule	30 –44 56	17	7.64	.022				

Abbreviations: MNI, peak voxel location in MNI space; *k*, cluster size; *t*, t‐statistic; *p*, *p*‐value.

### 3.2 Task‐specific activations: Shape versus roughness, roughness versus shape

**Figure 4:**
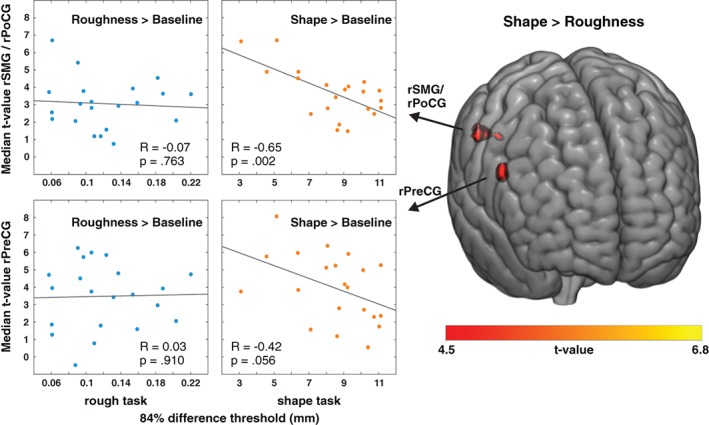
Correlation of behavioral performance with brain activation. The two clusters in the shape > roughness contrast are shown on a standard brain. These clusters were used to mask the roughness > baseline and shape > baseline contrast images on the first level. Median t values were extracted within each of the two clusters (areas of interest: rSMG/rPoCG, rPreCG) for each of the two contrasts (roughness > baseline, shape > baseline) and for every participant. The scatter graphs show the median *t*‐values of the areas of interest plotted against the 84% difference threshold of the roughness (left column) and the shape task (right column) of the respective participant (the lower the threshold, the better the performance) [Color figure can be viewed at http://wileyonlinelibrary.com]

The permutation test for the shape > roughness contrast yielded two significant clusters (see Table 3 and right part of Figure 4): One was located in the SMG (BA40) extending into the IPS and the PoCG and the other was located in the PreCG (ventral premotor cortex, vPMC/BA44) of the right hemisphere. We did not find a cluster of significantly higher activation in the roughness than in the shape task.

**Table 3:** Significant cluster activations in the right hemisphere in the shape > roughness contrast (cluster‐forming threshold = 4.54)

		MNI		*k*	*t*	*p* (cluster level)
	*x*	*y*	*z*			
Supramarginal gyrus and Postcentral gyrus	56	−24	42	46	6.76	.005
	44	−24	38		4.98	
Precentral gyrus	54	10	26	29	6.50	.012

### 3.3 Correlating behavioral performance and brain activation

A main goal of our study was to relate the results of the psychophysical task to the underlying brain activation. To this end, we masked the shape versus baseline and roughness versus baseline contrast images of each participant with the two significant clusters of the shape versus roughness contrast (SMG/PoCG and PreCG). This way we isolated *task‐specific* activations on the first level. Then, the median *t*‐value was extracted for each of the two regions of interest for either of the two task conditions yielding four median *t*‐values for every participant: For the SMG cluster for the shape versus baseline contrast, the SMG cluster for the roughness versus baseline contrast, the PreCG cluster for the shape versus baseline contrast, and the PreCG cluster for the roughness versus baseline contrast. We then correlated the median *t*‐values as a proxy for the brain activation in either task with the 84% difference threshold as a proxy for the behavioral performance of the participant in the respective discrimination task. The behavioral performance in the shape task correlated with the brain activation in the SMG, that is, the higher the SMG activation, the better the performance (i.e., the lower the threshold, see Figure 4, upper right panel; *R* = −.65, *p* = .002). Regarding the cluster in the PreCG, a trend was observed in the same direction (see Figure 4, lower right panel; *R* = −.42, *p* = .056). In contrast, performance in the roughness task did not correlate with brain activation in the SMG or PreCG (see Figure 4, left panels; *p* ≥ .763). Comparing the correlation coefficients, we found that the correlation within the SMG was significantly higher for the shape than for the roughness task (*Z*_20_ = 2.2, two‐tailed *p* = .031).

## 4 DISCUSSION

The present study examined how top‐down control changes the cortical processing of shape and roughness of manually explored objects and how these cortical changes relate to behavior. We presented the same carefully chosen sets of multidimensional objects under two different task instructions (judge either shape or roughness) and compared the brain activation patterns between the tasks. Activation of SMG and PreCG increased if object shape was task‐relevant. Importantly, brain activation in these areas predicted individual performance in the shape task. These results highlight the role of top‐down mechanisms in shape and roughness processing that lead to increased activation in SMG and PreCG which in turn can predict the shape discrimination behavior.

Contrasting the shape against the roughness task, we found two clusters of higher activation for shape than roughness but none for the reversed contrast. The identified shape clusters were located in the right hemisphere in the SMG extending into the anterior division of the IPS and the PoCG (BA 40) and the precentral gyrus (vPMC, BA 44). The SMG activation for the shape task correlated with the performance in the shape task (higher activation in shape > baseline, better performance in shape task) but not with that in the roughness task. A similar pattern of results was observed in the PreCG, but marginally missed the statistical significance. This result provides further evidence that the higher activation in SMG and PreCG is specific to shape perception.

The SMG and the vPMC have been associated with the manipulation of complex compared to simple shapes (Binkofski et al., 1999). Particularly, the importance of the SMG for the processing of (complex) haptic shape has been reported in previous research comparing the exploration of shape to that of other haptic features, such as roughness or curvature (Bodegård et al., 2001; O'Sullivan et al., 1994; Stoeckel et al., 2003). Bodegård et al. (2001) conducted a PET study in which objects were presented for haptic exploration that either differed in roughness, brush velocity, curvature (simple shape task), or oblongness (complex shape task). Only the exploration of complex shapes significantly increased the regional cerebral blood flow in the anterior part of the contralateral SMG whereas other somatosensory areas were activated irrespective of the haptic feature and were consequently interpreted as low‐level somatosensory areas. The SMG was suggested as the highest level in a hierarchy for the processing of complex shapes explored by touch.

As Bodegård et al. (2001), we find the anterior division of the SMG more active in the shape than in the roughness task, supporting their results. Crucially, the results of the present study suggest that the magnitude of activations in SMG and PreCG/BA 44 for a given explorative action depends on the task‐relevance of shape and, in turn, predicts individual shape discrimination performance. This extends the previous results by showing that activation in these areas does not merely reflect stimulus‐driven processes, such as exploring shapes but also entails top‐down controlled processes.

Other areas, that were previously reported to be potential neural correlates of haptic shape processing, failed to reach significance in our study when the shape task was contrasted against the roughness task. This can at least partly be explained by the design of our study. In the present study, two conditions were contrasted in which participants explored the same stimuli with the same exploration movement. Only the task was varied thus making either the shape or the roughness dimension of the object relevant. Most studies about haptic roughness and shape processing used a different approach in which different objects were explored across tasks. These additional differences between conditions also increase the likelihood of finding differences for accompanying brain activations. The only exception we are aware of is the study by Lederman, Gati, Servos, and Wilson (2001). They presented multidimensional objects that varied in shape (oblongness of ellipsoids), texture, and hardness, each on three levels. Participants explored the objects by a single squeezing movement and then verbally reported the level of the given feature. Task‐related activity was identified based on group correlation maps and did not reveal distinct areas for the processing of shape and texture but a common activation in the contralateral PoCG. With respect to the task design, eliminating stimulus‐driven activations from the processing of haptic information dramatically reduced activations, similar as for the task contrasts in the present study. This indicates strong commonalities between the cortical processing of shape and texture.

In order to relate our study to previous work, we contrasted each of the two tasks against a resting baseline, thus revealing areas indicative of lower‐level somatosensory processing. The baseline contrasts resulted in the network expected for haptic exploration, comprising the primary somatosensory and motor cortices. For both conditions we found large, bilateral activation clusters in the postcentral and precentral gyrus as well as in the cerebellum which is in line with previous findings (e.g., Stoeckel et al., 2003). The locations of the peak voxels (and also the extent of clusters) of the aforementioned areas were very similar across the tasks. This suggests that these areas play an important role in low‐level sensory processing of somatosensory stimuli which is dependent on the sensory input but not on task instruction. Areas that had been previously identified as distinctive regions for the processing of shape or roughness were found to be active in both conditions, for example, the insular cortices which has been associated with the processing of roughness, or the LOC which has been associated with the processing of shape, in particular related to mental imagery (Lacey, Tal, Amedi, & Sathian, 2009). The clusters in the right SPL, the inferior PoCG and the PO exceeded baseline activation and the cluster size threshold only in the shape task. The right SPL has been reported as a crucial region for kinesthetic attention involved in the discrimination of complex shapes (Stoeckel et al., 2004) which, while not significant when contrasted directly against the roughness task, fits well with the results of the present study. The activation of the PO in the shape rather than the roughness task is unexpected as this area has been previously associated with the processing of texture/roughness (Ledberg et al., 1995). Upon closer examination, an activation cluster of 13 voxels which did not pass the predefined cluster size threshold of >14 voxels, overlapped with the PO in the roughness versus baseline contrast. Overall, the results of the baseline contrasts are in line with previous studies of haptic shape and texture/roughness processing.

The question remains why we did not find activation in the roughness compared to the shape task. Interestingly, even studies comparing the processing of shape and roughness by using separate stimuli, that is, a task design focusing on selective processing of the two features, have been less consistent in identifying the neural correlates of micro‐ than macrogeometric features. Contrasting a length discrimination against a roughness task, O'Sullivan et al. (1994) identified brain areas specifically involved in the length/shape task (e.g., SMG) but not in the roughness task. The authors concluded that length discrimination requires more extensive processing and that roughness discrimination can be achieved by only a subset of areas that are also active in the length/shape task. A follow‐up study performed on the same data set (Ledberg et al., 1995), investigated whether specific areas within the PO can be activated by roughness but not by length discrimination. They observed ipsilateral PO activation in both tasks, while the roughness task additionally activated the contralateral PO. This activation overlapped with the activation in a somatosensory reaction time task except for one part of the contralateral PO which was exclusively activated by roughness perception. Based on that result it was unknown whether this part of PO was activated by the bottom‐up sensation of roughness or its relevance for the task. The lack of activation in the roughness > shape contrast in the present study suggests that roughness is processed automatically, irrespective of its relevance for the task, whereas the processing of haptic shape can be modulated by top‐down processes. Actually, response times from haptic search paradigms (e.g., find the rough stimulus among smooth ones) are consistent with the view that roughness is automatically processed, but shape is not (Lederman & Klatzky, 1997). However, our results do not necessarily exclude the benefits of directing attention to this feature on the behavioral level.

A caveat remains since the performance was worse in the shape than in the roughness task, indicating differences in task difficulty. Higher cognitive demands could lead to generally higher activation levels (Albanese, Duerden, Bohotin, Rainville, & Duncan, 2009) which might explain that we found activations in the shape versus roughness task but not vice versa. To account for task difficulty, we fitted a new GLM by adding the percent correct per task and exploration block as parametric regressor. To this end, activation associated with task difficulty should load on that regressor rather than on the task conditions. Previously found activations in the shape > roughness contrast persisted and were complemented by an activation located in the inferior frontal gyrus, a region associated with selective attention in haptic perception (Binkofski et al., 1999; Reed et al., 2004; Reed, Klatzky, & Halgren, 2005; Stoeckel et al., 2003) and fine motor control (Liakakis, Nickel, & Seitz, 2011). Still, no activation was found in the roughness > shape contrast, thus rendering task difficulty as explaining factor unlikely.

In summary, we were able to identify clusters in the SMG and in the PreCG associated with task‐relevance of the shape feature and thus top‐down modulation. Additionally, brain activation within these clusters correlated with behavioral performance in the shape but not in the roughness discrimination task. Importantly, our design controlled for stimulus‐driven activations by presenting the same objects across tasks that were explored by the same movement. Thus, we extend the results of previous studies by showing that the identified clusters are mediated by top‐down control mechanisms associated with the processing of shape rather than by bottom‐up sensory stimulation. We did not find higher activation for the reversed contrast, indicating that the microgeometric property roughness is automatically processed even if irrelevant for a given task.

## CONFLICT OF INTEREST

No conflict of interest has been declared by the authors.

## Supporting information


**Figure S1** Psychometric functions of the individual subjects’ performance in the shape and roughness task

**Figure S2** First level design matrix for one subject

## Data Availability

The data and the analyses scripts of this study are publicly available at https://doi.org/10.5281/zenodo.3267417.
