# Supplementary material for: Neural correlates of top‐down modulation of haptic shape versus roughness perception
Source: Hum Brain Mapp. 2019 Aug 20;40(18):5172–84. doi: 10.1002/hbm.24764 (PMC6864886; doi:10.1002/hbm.24764)
Supplement: Supplementary file 1 — Figure S1 Psychometric functions of the individual subjects’ performance in the shape and roughness task [file HBM-40-5172-s001.pdf]

Psychometric functions of the individual subjects‘ performance in the shape and roughness task

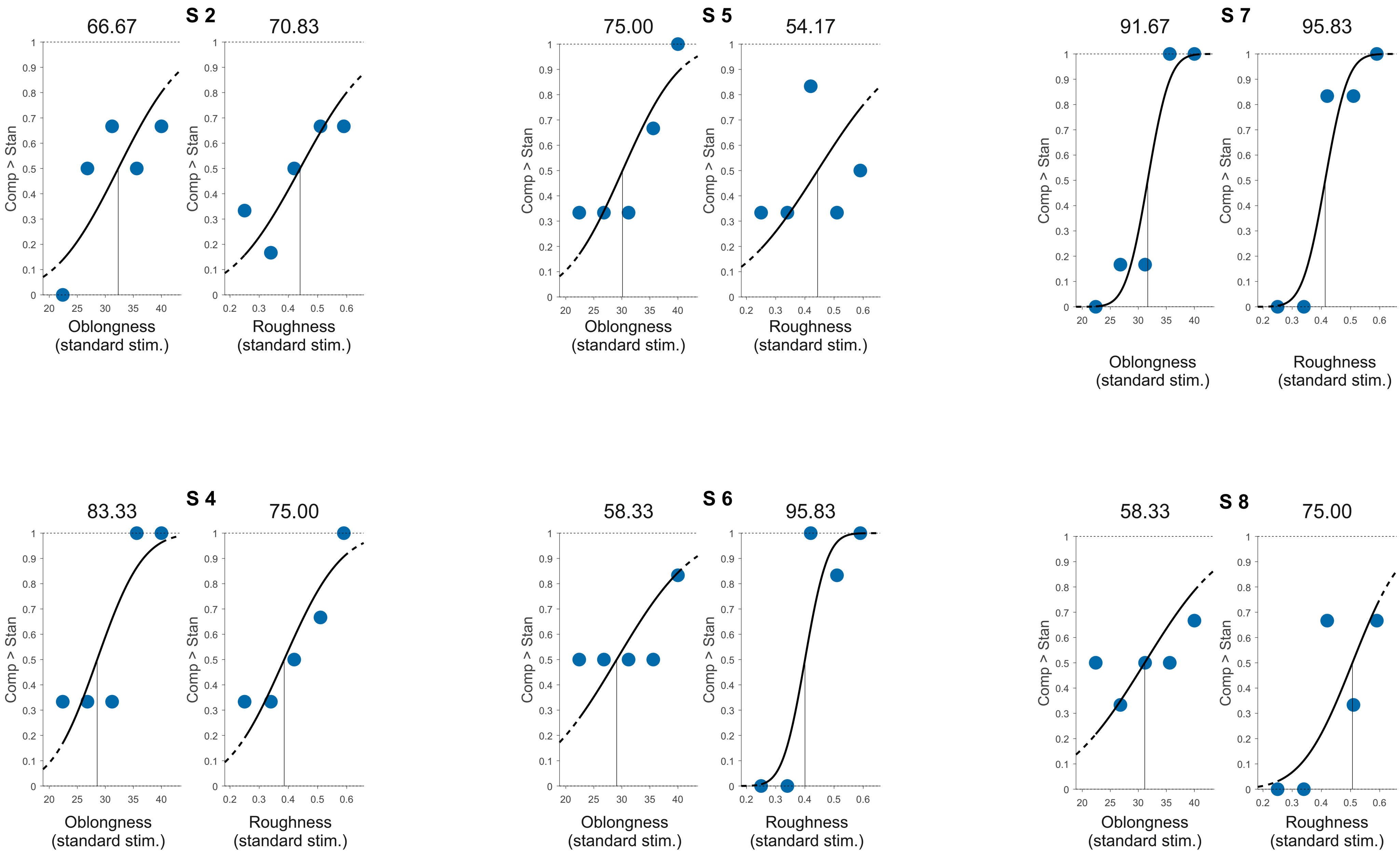

Psychometric functions of the individual subjects‘ performance in the shape and roughness task

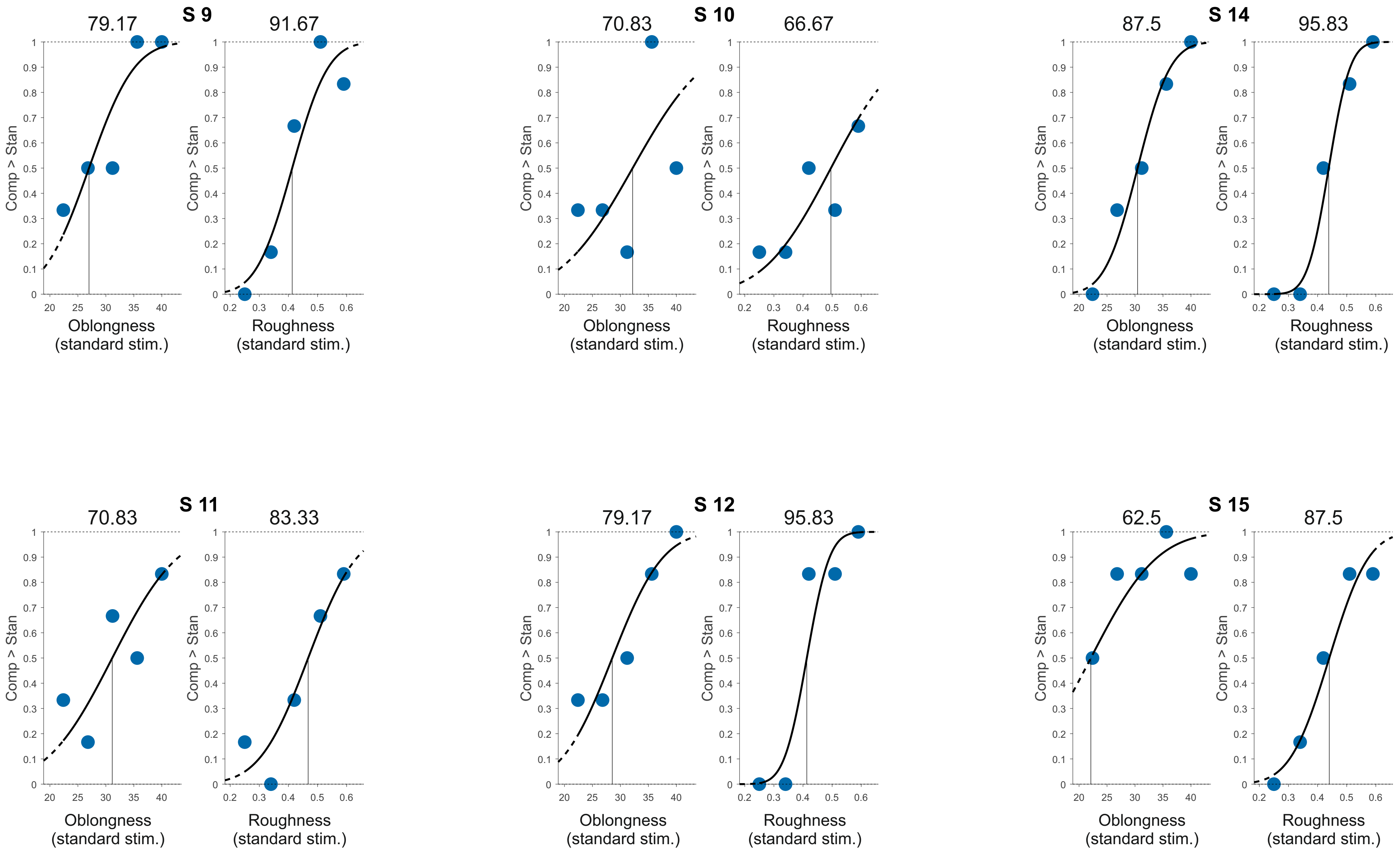

Psychometric functions of the individual subjects‘ performance in the shape and roughness task

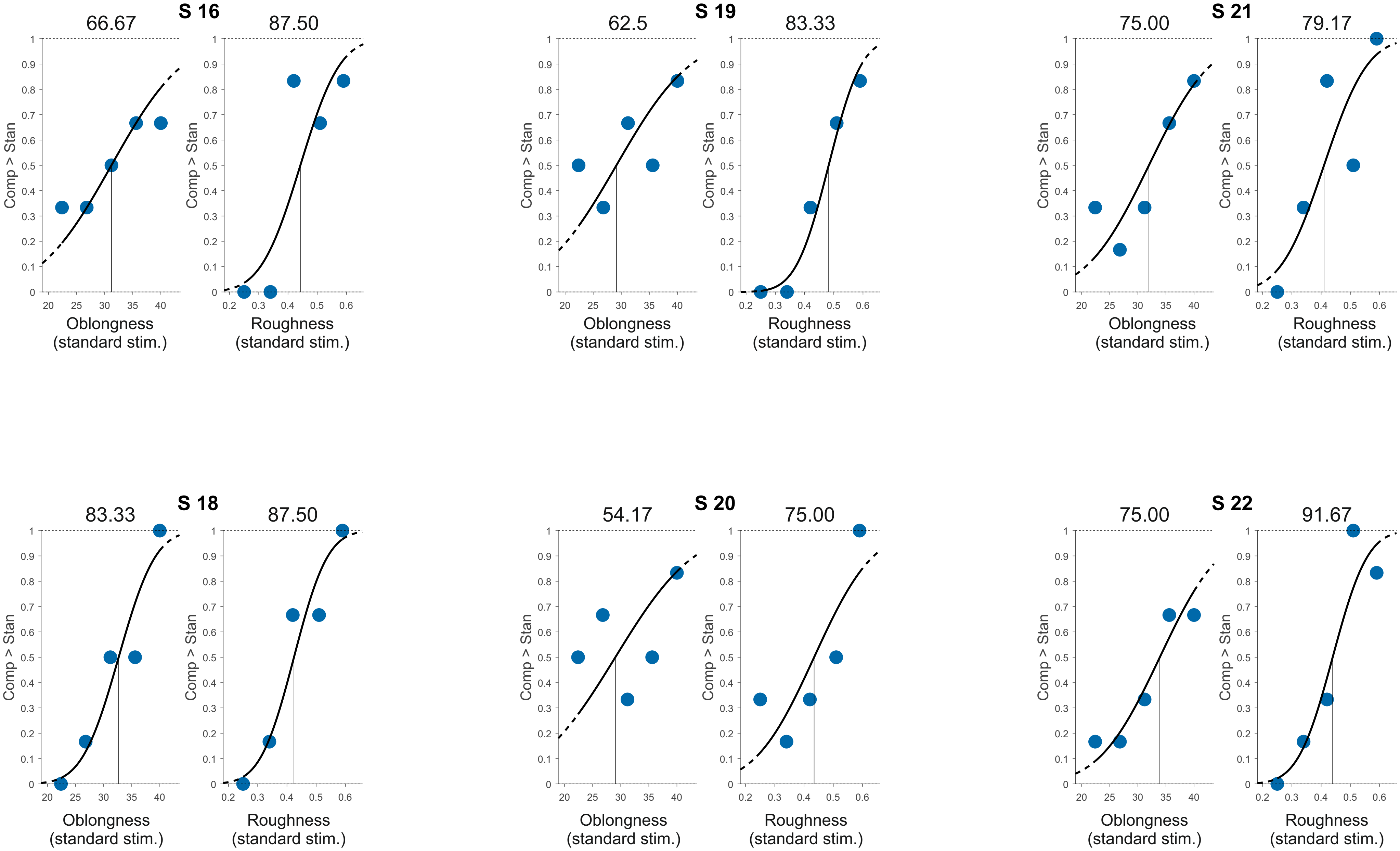

Psychometric functions of the individual subjects‘ performance in the shape and roughness task

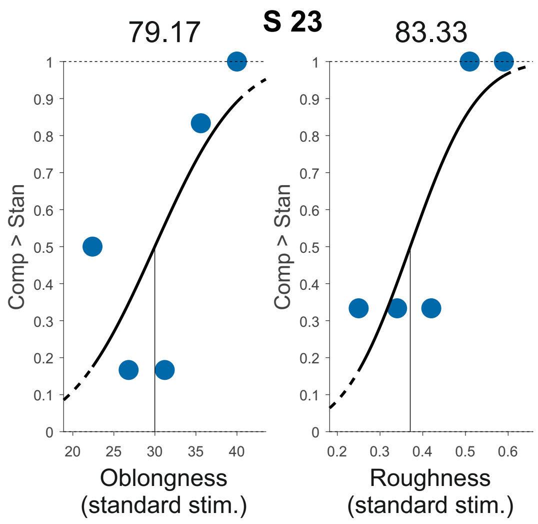

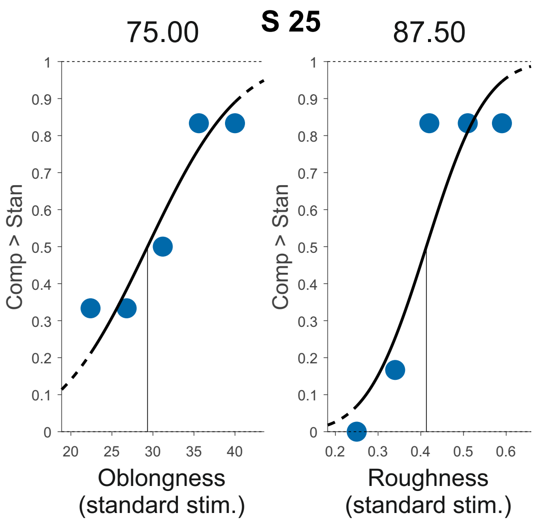

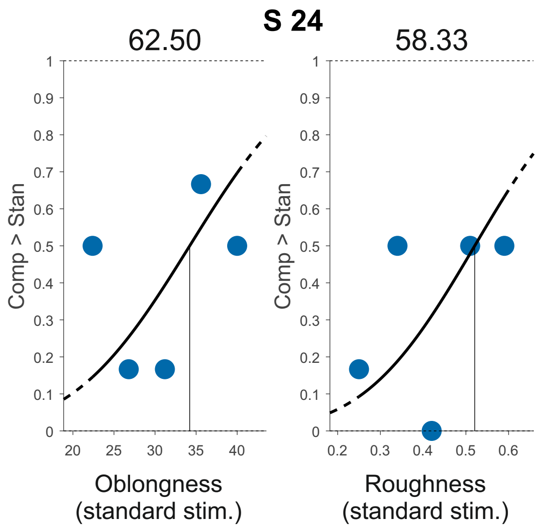

Top: Percent correct

Left panels: Shape task

Right panels: Roughness task

y-axes: Proportion of comparison stimulus judged longer/rougher than standard stimulus

x-axes: Oblongness of longer side of the shape standard stimulus (left panels) / Roughness (right panels) of the roughness standard stimulus

	84% Difference Threshold	
**Subject**	**Shape task**	**Roughness task**
2	9.06	0.19
4	6.38	0.15
5	8.01	0.22
6	10.74	0.06
7	3.08	0.06
8	11.14	0.14
9	6.36	0.10
10	10.16	0.18
11	9.21	0.13
12	7.09	0.06
13	11.59	0.08
14	4.57	0.06
15	9.27	0.11
16	10.12	0.11
18	5.14	0.09
19	10.40	0.09
20	11.04	0.16
21	8.75	0.12
22	8.53	0.09
23	8.07	0.12
24	11.14	0.20
25	8.62	0.11
