# Supplementary material for: Neural correlates of top‐down modulation of haptic shape versus roughness perception
Source: Hum Brain Mapp. 2019 Aug 20;40(18):5172–84. doi: 10.1002/hbm.24764 (PMC6864886; doi:10.1002/hbm.24764)
Supplement: Supplementary file 2 — Figure S2 First level design matrix for one subject [file HBM-40-5172-s002.pdf]

First Level Design Matrix for one Subject

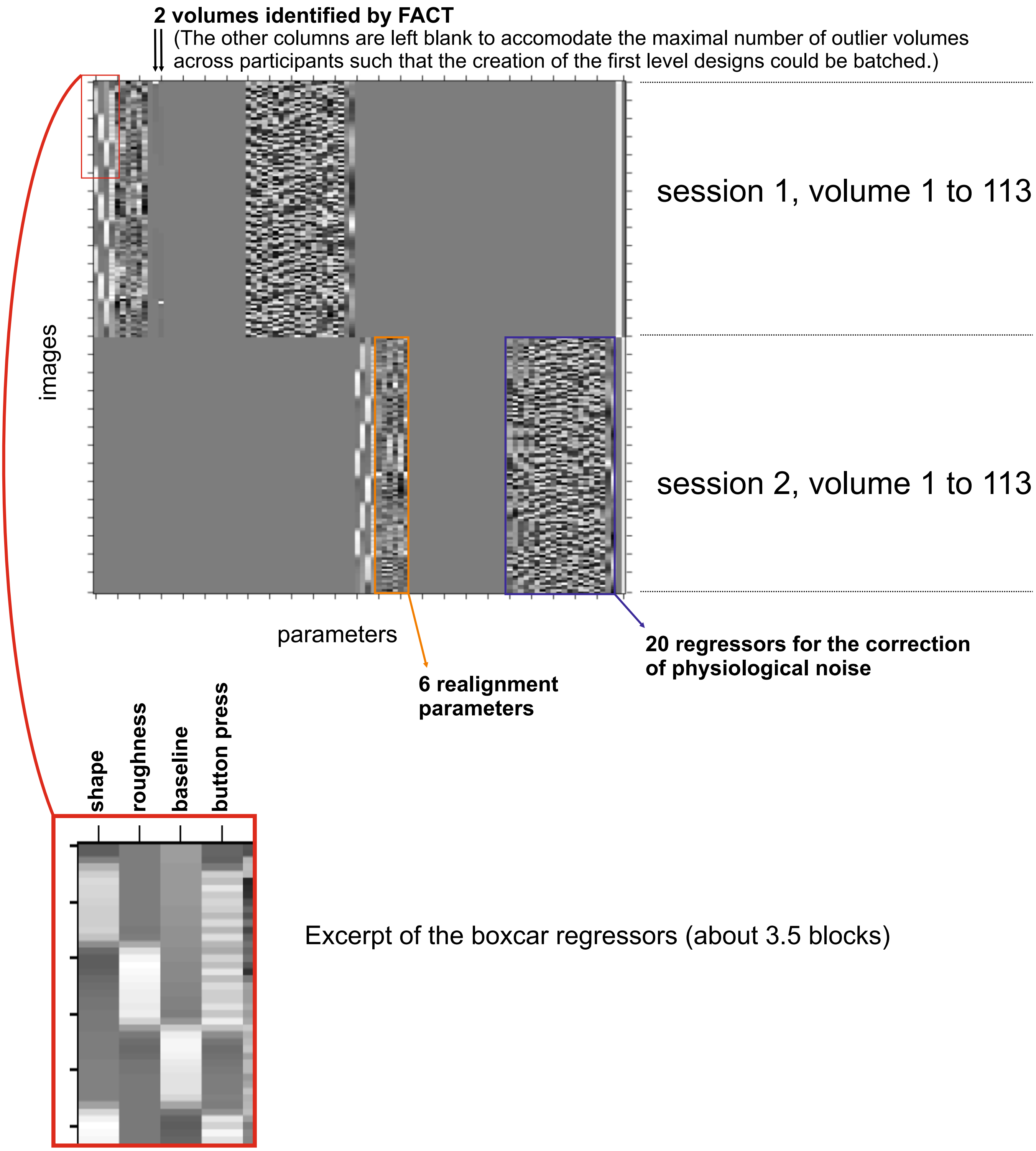

Excerpt of the boxcar regressors (about 3.5 blocks)
